# First Baby Born in Brazil after Simultaneous Diagnosis through Non-Invasive and Conventional PGT-A

**DOI:** 10.1055/s-0041-1736302

**Published:** 2021-12-06

**Authors:** Marcos Iuri Roos Kulmann, Márcia Riboldi, Carolina Martello, Adriana Bos-Mikich, Gerta Frantz, Caroline Dutra, Luiza Mezzomo Donatti, Norma Oliveira, Nilo Frantz

**Affiliations:** 1 Nilo Frantz Medicina Reprodutiva, Porto Alegre, RS, Brazil; 2 Igenomix Brasil, São Paulo, SP, Brazil; 3 Department of Morphological Sciences, Instituto de Ciências Básicas da Saúde, Universidade Federal do Rio Grande do Sul, Porto Alegre, RS, Brazil

**Keywords:** niPGT-A, spent culture media, PGT-A, trophectoderm biopsy, blastocyst, niPGT-A, meio de cultivo condicionado, PGT-A, biópsia de trofectoderma, blastocisto

## Abstract

Non-invasive preimplantation genetic testing for aneuploidies (niPGT-A) aiming to assess cell-free embryonic DNA in spent culture media is promising, especially because it might overcome the diminished rates of implantation caused by the inadequate performance of trophectoderm (TE) biopsy. Our center is part of the largest study to date assessing the concordance between conventional PGT-A and niPGT-A, and we report here the delivery of the first baby born in Brazil using niPGT-A. The parents of the baby were admitted to our center in 2018. They did not present history of infertility, and they were interested in using in vitro fertilization (IVF) and PGT-A in order to avoid congenital anomalies in the offspring. A total of 11 (3 day-5 and 8 day-6) expanded blastocysts were biopsied, and the spent culture media (culture from day-4 to day-6) from 8 day-6 blastocysts were collected for niPGT-A. Overall, 7 embryos yielded informative results for trophectoderm (TE) and media samples. Among the embryos with informative results, 5 presented concordant diagnosis between conventional PGT-A and niPGT-A, and 2 presented discordant diagnosis (1 false-positive and one false-negative). The Blastocyst 4, diagnosed as 46, XY by both niPGT-A and conventional PGT-A, was warmed up and transferred, resulting in the birth of a healthy 3.8 kg boy in February 2020. Based on our results and the recent literature, we believe that the safest current application of niPGT-A would be as a method of embryo selection for patients without an indication for conventional PGT-A. The approximate 80% of reliability of niPGT-A in the diagnosis of ploidy is superior to predictions provided by other non-invasive approaches like morphology and morphokinetics selection.

## Resumo

Abordagens para o teste genético pré-implantacional não-invasivo para aneuploidias ( *non-invasive preimplantation genetic testing for aneuploidies* , niPGT-A, em inglês) com o objetivo de avaliar o DNA embrionário livre são promissoras, especialmente porque estas podem reverter as menores taxas de implantação causadas por inadequada biópsia de trofectoderma (TE). Nesse contexto, nosso centro é parte do maior estudo atual que avalia as taxas de concordância entre PGT-A convencional e niPGT-A, e relatamos aqui o nascimento do primeiro bebê brasileiro após niPGT-A. Os pais do bebê foram admitidos no nosso centro em 2018. Eles não apresentavam histórico de infertilidade, e estavam interessados em utilizar os tratamentos de fertilização *in vitro* (FIV) e PGT-A para evitar anomalias congênitas na progênie. Um total de 11 blastocistos expandidos (3 do dia-5 e 8 do dia-6) foram submetidos a biópsia, e os meios de cultivo condicionados (cultivo do dia-4 ao dia-6) de 8 blastocistos do dia-6 foram coletados para niPGT-A. No total, resultados informativos para as amostras de TE e dos meios foram obtidos para sete embriões. Entre os embriões com resultado informativo, 5 apresentaram diagnóstico concordante entre PGT-A convencional e niPGT-A, e 2 apresentaram diagnóstico discordante (1 falso positivo e 1 falso negativo). O Blastocisto 4, diagnosticado como 46, XY por ambos niPGT-A e PGT-A convencional, foi desvitrificado e transferido, o que resultou no nascimento de um menino saudável, que pesava 3,8 kg, em fevereiro de 2020. Com base em nossos resultados e literatura contemporânea, acreditamos que a aplicação atual mais segura do niPGT-A seria como método de seleção embrionária para pacientes sem indicação ao PGT-A convencional. A confiabilidade aproximada de 80% do niPGT-A para determinação da ploidia ainda é superior àquela obtida com abordagens não invasivas, como seleção morfológica ou morfocinética.

## Introduction

The technology for in vitro fertilization (IVF) has evolved greatly towards the achievement of higher success rates, and current state-of-the-art laboratories apply extended culture to blastocyst stage, vitrification, and time-lapse incubators, for instance. 1 One main issue in reproductive medicine that these technological advances cannot overcome is advanced maternal age (AMA), which reduces implantation rates due to the higher frequency of aneuploidies of meiotic origin linked to age. 2

To address this matter, preimplantation genetic testing for aneuploidies (PGT-A), performed through a trophectoderm (TE) biopsy and next generation sequencing (NGS), can be applied. It has been shown to increase implantation rates in AMA patients when a euploid blastocyst is available for transfer, to decrease miscarriage rates, to be cost-effective, and to shorten treatment time. 3 4 5 Nevertheless, recent reports 6 7 of deliveries from mosaic/aneuploid blastocysts call into question the reliability of a TE biopsy to estimate the inner-cell-mass (ICM) ploidy. In addition, TE biopsy is an invasive procedure that requires expensive specific equipment and highly-trained embryologists. As a consequence, the biopsy can impair implantation rates if not properly performed. 8 9 In this sense, a non-invasive approach for chromosome screening would be preferred.

Non-invasive PGT-A (niPGT-A) approaches aiming to assess cell-free embryonic DNA in spent culture media are promising. However, several studies 10 11 12 13 14 have reported strikingly variable concordance rates, ranging from 30.3% to 85.7%, between media and TE samples, which undermines the clinical applicability of this technology. Interestingly, Huang et al. 15 have achieved a concordance of 93.8% when comparing media and whole embryo results. All of these previous studies have employed additional manipulation on analyzed embryos, like vitrification or assisted hatching, prior to the collection of spent culture media, approaches that would not be applied during an IVF cycle with niPGT-A. In this sense, Rubio et al. 16 have developed a pilot study aiming to assess the concordance between media and TE samples from blastocysts that were not submitted to those additional manipulations. As a result, they have observed an 84% concordance for day-6 or -7 blastocysts.

To fully evaluate this protocol, Rubio et al. 17 have expanded this approach to a prospective multicenter study, including our center as the Brazilian representative. Until this moment, the analysis of TE and media samples from 1,301 blastocysts (from 8 IVF centers) has provided an overall concordance of 78.2%, 17 and the delivery of the first baby born in Brazil after niPGT-A.

## Case Description

A 35-year-old woman was admitted to our center in 2018 for a first medical consultation accompanied by her 37-year-old husband. The couple did not present history of infertility, and they were interested in using IVF and PGT-A in order to avoid congenital anomalies in the offspring. The wife presented a normal antral follicle count, a serum concentration of anti-Müllerian hormone of 2.65 ng/mL, and a normal body mass index (BMI = 20.2 kg/m ^2^ ). The husband presented normal sperm concentration, motility and morphology, and a BMI of 28.4 kg/m ^2^ , which is suggestive of overweight. The sperm DNA fragmentation index of 39% indicated poor sperm quality. The couple decided to undergo IVF and enroll in our prospective study assessing the concordance between PGT-A from TE biopsy and niPGT-A from cell-free DNA in spent culture media.

The patient underwent a gonadotropin-releasing hormone (GnRh) antagonist (Orgalutran) regimen, with application from days 7 to 10 of the stimulation. Recombinant follicle-stimulating hormone (FSH; Puregon) was applied from days 1 to 5 on a daily dose of 275 IU, and human menopausal gonadotropin (hMG; Menopur), from days 5 to 10 on a daily dose of 225 IU. When the dominant follicle reached 18 mm, the patient received a single dose of human chorionic gonadotropin (hCG; Choriomon). All laboratory material, media, oil and pipettes used during the treatment were exclusively manipulated by embryologists wearing gloves, caps, and masks to avoid external DNA contamination. Oocyte pick-up was performed 36 hours after the administration of hCG, and it resulted in the collection of 21 cumulus-oocyte complexes. On the date of the aspiration, the seminal sample had a concentration of 60 million sperm cells/mL, and 65% of progressive motility. After extensive elimination of corona-cumulus cells to prevent maternal contamination, 18 metaphase-II (MII) oocytes were identified and inseminated by intracytoplasmic sperm injection (ICSI). After 18 hours of the ICSI, a morphological assessment indicated 18 fertilized oocytes presenting 2 pronuclei and the extrusion of the second polar body.

The embryos were individually cultured from the pronuclear stage until day 4 in 25 µL droplets of Continuous Single Culture Complete medium (CSCM-C) in low oxygen (5%) conditions (G185 incubator, K-Systems). At day 4, each embryo was washed in 6 droplets and transferred to a 10-µL droplet of CSCM-C medium using an individual stripper pipette. A total of 11 (3 day-5 and 8 day-6) expanded blastocysts were biopsied. For this purpose, laser zona opening was performed at the time of biopsy, and 5 to 10 TE cells from the expanded blastocyst were harvested as previously described. 18 After the biopsy, the blastocysts were transferred to a new droplet of medium before vitrification. The spent culture media (culture from days 4 to 6) from 8 day-6 blastocysts (blastocysts 4 to 11) were collected for niPGT-A. Blastocysts 1, 2 and 3 were biopsied in day 5, and their media samples were not analyzed due to the previous low concordance between PGT-A and niPGT-A results observed for day-5 blastocysts. 16 Both conventional PGT-A and niPGT-A were performed through NGS technology.

Overall, 7 embryos yielded informative results for both TE and media samples, and 1 embryo yielded a non-informative result due to amplification failure from the TE biopsy ( Table 1 ). Among the embryos with informative results, 5 presented concordant diagnosis, and 2, discordant diagnosis (1 false-positive and 1 false-negative). Blastocysts 4, 6 and 9 presented total concordance between TE and media samples. Blastocyst 7 was diagnosed as aneuploid by both approaches; however, it exhibited a complementary pattern (-18q in TE and +18 in medium). Additionally, blastocyst 5 presented partial concordance, since the monosomy of chromosome 21 was detected only through niPGT-A. Blastocyst 8 was diagnosed as aneuploid based on the TE sample, and as euploid based on the medium sample (false-negative). On the other hand, blastocyst 10 was diagnosed as euploid based on the TE sample, and as aneuploid based on the medium sample (false-positive).

**Table 1:** PGT-A and niPGT-A results

Blastocyst	PGT-A	niPGT-A	
**4**	46, XY	46, XY	Total concordance
**5**	45, -20, XX	45, +20, -21, X0	Partial concordance
**6**	44, -11, -14, XY	44, -11, -14, XY	Total concordance
**7**	46, -18q, XY	47, +18, XY	Partial concordance
**8**	46, -6, +21, XY	46, XY	False-negative
**9**	47, +10, XY	47, +10, XY	Total concordance
**10**	46, XX	51, +1, +4, +10, +21, +22, XX	False-positive
**11**	Noninformative results		

Abbreviations: niPGT-A, non-invasive preimplantation genetic testing for aneuploidies; PGT-A, preimplantation genetic testing for aneuploidies.

**Fig. 1:**
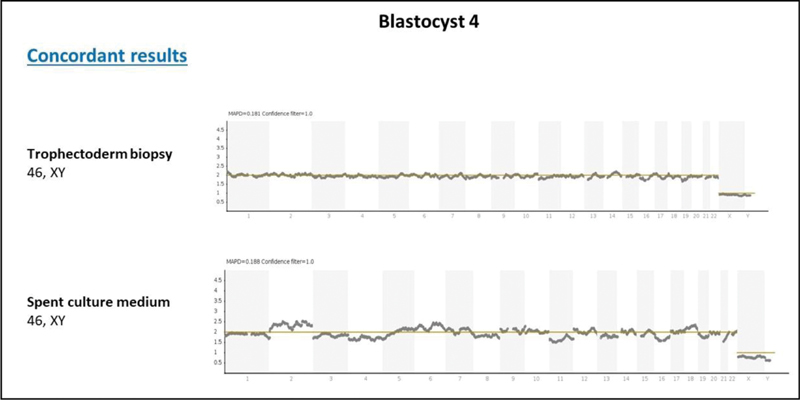
Profiles representing the diagnosis of blastocyst 4 by conventional PGT-A and niPGT-A.

In May 2019, the couple decided to undergo their first frozen embryo transfer. The endometrium was prepared with a daily administration of estradiol valerate (Primogyna) starting on the second day of the menstrual period. At the 12th day of preparation, the endometrium had a thickness of 7.5 mm, presenting a triple-line pattern. From this moment on, the patient received a daily dose of intravaginal progesterone (Utrogestan) for five days before the transfer. Blastocyst 4, diagnosed as 46, XY by both niPGT-A from spent culture media and conventional PGT-A from TE biopsy ( Fig. 1 ), was warmed up and transferred. After 10 days, beta-hCG quantification yielded a positive result, and pregnancy developed until 40 weeks with the birth of a healthy 3.8 kg male newborn in February 2020.

## Discussion

Along with the advances in molecular techniques, the clinical widespread application of niPGT-A is close to a reality in IVF treatments. We report here the first baby born after niPGT-A in Brazil as part of a multicenter prospective study assessing the concordance between diagnosis provided by TE biopsy and spent culture media.

In this particular case, the couple was not infertile, and the wife was not of advanced age, so they did not have a clinical indication for either IVF or PGT-A, and they were counseled accordingly. Despite the recommendations, they decided to undergo such treatments due to their personal beliefs and experiences regarding the occurrence of congenital abnormalities. With the development of niPGT-A technology, we must discuss its clinical indications and the ethical implications of adding a new diagnostic tool in the absence of a health problem. Differently from conventional PGT-A, niPGT-A has the advantage of being completely non-invasive, just like the morphology or morphokinetics evaluations. None of these non-invasive selection methods causes harm to the embryo, and they have the same aims: to strengthen embryo selection and decrease the number of embryo transfers to achieve a live birth.

Three biopsied blastocysts from the couple presented total concordance, while two presented partial concordance between TE and media samples, which is in agreement with the results of the study by Rubio et al. 17 In their interim analysis, 17 8 different centers obtained an overall concordance of 78.2% (range: 72.5% to 86.3%), a sensitivity of 81.7% (range: 76.5% to 91.3%) and a specificity of 77.4% (range: 64.7% to 87.5%). Interestingly, concordance was not affected by the center where the testing was conducted, or by the incubator model or culture media used, which supports the reproducibility of the protocol. For the analysis of the blastocysts, the spent culture media in contact with the embryo from day 4 to days 6/7 were collected. This protocol was based on previous studies conducted by Igenomix that reported inferior concordance rates when using media from day 3 to 5 (33.3%) 12 or day 4 to 5 (63.0%). 16

The need to extend the blastocyst culture until days 6/7 to obtain reasonable concordance rates between TE and media samples is a drawback of niPGT-A that must be cautiously analyzed. While it is generally assumed that the implantation potential of untested day-6/7 blastocysts is inferior to that of day-5 blastocysts, 19 20 21 22 23 24 the case for the transfer of euploid blastocysts is still under debate. 25 26 In addition, further studies should assess if blastocysts formed only on days 6/7 and those formed on day 5 and maintained in culture until days 6/7 present different potentials of implantation.

One important obstacle for obtaining low false-negative rates from niPGT-A is the maternal contamination from corona-cumulus cells that were not completely eliminated before ICSI. During our validation process, we noticed inferior concordant rates for patients with cells adhered to the zona pellucida even after oocyte denudation (personal observation). To solve this problem, we increased the concentration of hyaluronidase solution, established an incubation of 20 minutes between chemical and mechanical denudations, and removed all corona-cumulus cells during the mechanical denudation. Additionally, the use of gloves, caps, masks and exclusive laboratory materials/reagents is crucial to avoid degradation or contamination of media samples.

The transferred blastocyst 4 was diagnosed as 46, XY by both conventional PGT-A and niPGT-A, and resulted in the delivery of a healthy newborn. In this sense, a previous study by Rubio et al. 16 suggested higher ongoing implantation rates for blastocysts with a concordant euploid diagnosis (52.9%) in comparison to embryos diagnosed as euploid by the TE biopsy and aneuploid by the spent culture media (16.7%). Additionally, Huang et al. 15 reported a concordance between media and whole blastocysts (93.8%) higher than that of TE and whole blastocysts (82%) for a set of 50 embryos, suggesting that niPGT-A would be less biased by the issue of embryonic mosaicism. However, in a recent study, Rubio et al. 17 found concordances of 84.4% and 87.5% between media and ICM and TE and ICM respectively, for a set of 80 embryos.

Based on the concordance rate achieved in our own experience, niPGT-A is still not ready to replace conventional PGT-A, especially for patients who are at risk of having aneuploid pregnancies (such as AMA patients). In this context, the current feasible application of niPGT-A would be as an embryo-selection method for patients without indication for conventional PGT-A. The reliability of ∼ 80% of niPGT-A in the diagnosis of ploidy diagnosis is superior to that provided by morphological 18 27 or morphokinetics evaluations. 28 The benefits of niPGT-A as an embryo-selection method should be demonstrated through randomized controlled trials.
